# Quality assurance of add-on testing in plasma samples: stability limit for 29 biochemical analytes

**DOI:** 10.11613/BM.2024.020704

**Published:** 2024-04-15

**Authors:** Francisco J. Illana, Álvaro García-Osuna, María Sospedra, Rosa Ferrer, Cecília Martínez-Brú, Leonor Guiñón

**Affiliations:** 1 Biochemistry Department, Hospital de la Santa Creu i Sant Pau, Barcelona, Spain; 2 Institut de Recerca Sant Pau (IR SANT PAU), Barcelona, Spain; 3 Core Laboratory, Hospital de la Santa Creu i Sant Pau, Barcelona, Spain; 4 Laboratories Quality Department, Hospital de la Santa Creu i Sant Pau, Barcelona, Spain

**Keywords:** automation, laboratory organization and management, sample stability, add-on testing, quality assurance

## Abstract

**Introduction:**

Clinical laboratories should guarantee sample stability in specific storage conditions for further analysis. The aim of this study is to evaluate the stability of plasma samples under refrigeration for 29 common biochemical analytes usually ordered within an emergency context, in order to determine the maximum allowable period for conducting add-on testing.

**Materials and methods:**

A total of 20 patient samples were collected in lithium heparin tubes without gel separator. All analyses were performed using Alinity systems (Abbott Laboratories, Abbott Park, USA) and samples were stored at 2-8 °C. Measurements were conducted in primary plasma tubes at specific time points up to 48 hours, with an additional stability study in plasma aliquots extending the time storage up to 96 hours. The stability limit was estimated considering the total limit of change criteria.

**Results:**

Of the 29 studied parameters, 24 demonstrated stabilities within a 48-hour storage period in primary plasma tubes. However, five analytes: aspartate aminotransferase, glucose, lactate dehydrogenase, inorganic phosphate and potassium evidenced instability at different time points (7.9 hours, 2.7 hours, 2.9 hours, 6.2 hours and 4.7 hours, respectively). The stability study in plasma aliquots showed that all parameters remained stable for 96 hours, except lactate dehydrogenase, with a stability limit of 63 hours.

**Conclusions:**

A reduced stability of primary plasma samples was observed for five common biochemical analytes ordered in an emergency context. To ensure the quality of add-on testing for these samples, plasma aliquots provide stability for a longer period.

## Graphical abstract

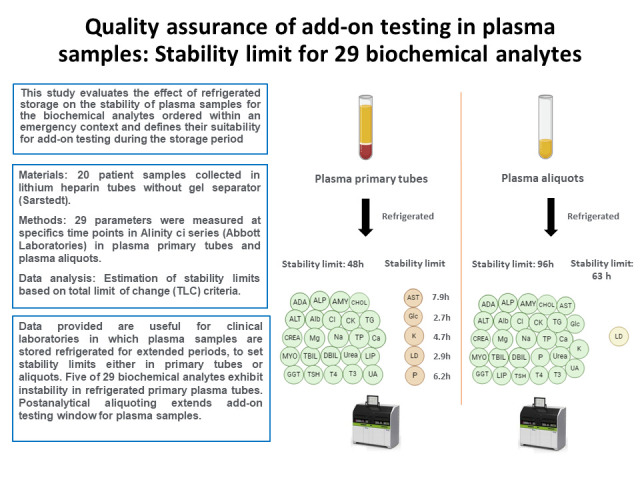

## Highlights

• Aspartate aminotransferase, glucose, lactate dehydrogenase, phosphorus and potassium showed instability in refrigerated primary plasma tubes

• All analytes, except lactate dehydrogenase, remained stable in refrigerated plasma aliquots

• Postanalytical aliquoting extends add-on testing time window for plasma samples

## Introduction

Storing serum or plasma samples after the completion of the requested analysis is a standard practice in clinical laboratories. The specimen storage conditions play a pivotal role in maintaining the integrity of samples after the initial analysis. Add-on tests are frequently requested for clinical samples already present in the laboratory, typically in response to the results of the initial tests. This approach not only spares patients from additional venepuncture but also enhances laboratory efficiency and reduces turnaround time for the requested tests (*1*).

Due to the critical impact of analytical results on patient safety, clinical laboratory professionals bear the responsibility of ensuring the stability of samples throughout their storage period, thereby safeguarding the quality of analytical results for subsequent add-on tests. In this sense, the new edition of the ISO 15189:2022 standard states in section 7.2. the specific requirements to establish time limits for requesting additional examinations (*2*).

At our hospital, the majority of tests (more than 75%) are performed within the biochemistry area of the core laboratory, accounting for approximately 1000 orders daily (70% routine samples, 30% STAT samples). In particular, 5% of the orders correspond to add-on testing, with the Emergency Department being the main source of these orders (50% approximately).

Recently, the biochemistry area in our laboratory underwent automation, integrating a refrigerated storage module with capacity for 10,000 tubes connected with the preanalytical and analytical area *via* a track. Before automation, the postanalytical storage was conducted using different procedures to ensure the stability of the analytes. After the completion of analysis, routine samples were stored refrigerated for 7 days in the serum separator tubes with gel; while for STAT samples aliquots of plasma anticoagulated with lithium-heparin were stored frozen for 2 days.

In our core laboratory, routine and STAT samples are managed simultaneously, optimizing the utilization of personnel and reagents. With the automation of the area raised the integration of the postanalytical phase for both, routine and STAT samples, through the automated sorting and storing of primary tubes. For routine samples, analyte stability during the storage period was guaranteed by previous studies reported in the literature (*3*, *4*). However, incomplete, confusing and conflicting evidence was found regarding analyte stability in plasma, both in the reagent information provided by the *in vitro* diagnostic company and in the stability studies published to date (*5*, *6*).

The present study evaluates the impact of refrigerated storage (2-8 °C) on the stability of plasma samples over durations ranging from 2 to 48 hours in primary tubes, with an extended evaluation to 96 hours for aliquots. This assessment focuses on the most common biochemical analytes and aims to determine their suitability for add-on requests during the storage period.

## Materials and methods

### Materials

A total of 20 leftover plasma samples, collected in lithium heparin tubes without gel separator (Sarstedt, Nümbrecht, Germany) were selected from anonymous patients in the emergency department in September and October of 2023. Prior to analysis, the samples underwent centrifugation at 1900xg for 15 minutes in a Kubota 4000 (KUBOTA, Tokio, Japan). Sample interference indices for hemolysis, icterus and lipemia were measured using the Alinity c system (HIL Saline Protocol) to confirm the absence of interferences.

Additionally, to maintain the add-on testing timeframe established in the laboratory prior to automation, we planned to perform an extra study on plasma aliquots for any analyte with a stability limit below 48 hours in plasma primary tubes.

Ethical approval for the study was granted by the Clinical Research Ethics Committee of our hospital (IIBSP-EMB-2023-78).

### Methods

Adenosine deaminase (ADA), alanine aminotransferase (ALT), albumin (Alb), alkaline phosphatase (ALP), amylase (AMY), aspartate aminotransferase (AST), direct bilirubin (DBIL), total bilirubin (TBIL), calcium (Ca), chloride (Cl), total cholesterol (CHOL), creatinine (CREA), creatinine kinase (CK), glucose (Glc), gamma-glutamyltransferase (GGT), lactate dehydrogenase (LD), lipase (Lip), magnesium (Mg), inorganic phosphate (Phos), potassium (K), sodium (Na), total protein (TP), triglycerides (TG), urea and uric acid (UA) were measured using the Alinity c system (Abbott Laboratories, Abbott Park, USA). Thyroid stimulating hormone (TSH), triiodothyronine-free (fT3) and thyroxine-free (fT4) were measured using the Alinity i system (Abbott Laboratories, Abbott Park, USA). All used reagents were Alinity original reagents (Abbott Laboratories, Abbott Park, USA), with the exception of ADA (Biosystems, Barcelona, Spain) and Lip (Architect reagent 7D80, Abbott Laboratories, Abbott Park, USA) adaptations.

For each sample collected in plasma primary tubes, 29 parameters were measured at specific time points: 0, 2, 4, 6, 8, 12, 24 and 48 hours. Exceptionally, some time points were not tested (NT) in plasma primary tubes due to technical reasons.

For parameters exhibiting reduced stability in plasma primary tubes, an extended study in plasma aliquots was conducted at 24, 48, 72 and 96 hours. For the remaining parameters stability was only checked at 96 hours, then stability in plasma aliquots at 24, 48 and 72h was not studied.

At each point, the handling of plasma samples at room temperature was limited to a maximum of 15 minutes. After each measurement, samples were sealed and stored in a refrigerator (2-8 °C).

### Data analysis

For each patient sample, relative differences were estimated as percentage differences (PD%) between basal measurement values (time 0) and the values at the different time points selected, according to the formula: PD% = [(t_x_ - t_0_) / t_0_] x 100, where t_x_ is the concentration of an analyte at a specific time point x and t_0_ is the concentration of an analyte at time 0. Next, for each time point, the mean of the PD% was calculated. Maximum permissible differences between each time point and time 0 were defined based on the Total Limit of Change (TLC), according to the following equation: TLC = ± √(1.65*CVa)^2^ + (0.5*CVb)^2^, where CVa is the analytical variability of the measurement procedure in the last 6 months and CVb is the intraindividual biological variability obtained from the European Federation of Clinical Chemistry and Laboratory Medicine (EFLM) biological variation database (*5*, *7*, *8*).

Finally, when the mean of the PD% exceeded TLC at any time point, the stability limit (SL) was estimated by means of the instability equation through a regression analysis, in accordance with EFLM Working Group Preanalytical Phase recommendations as follows: SL = TLC / β, where β is the slope of the instability equation for each analyte (*9*). Stability limit was established at the storage time (in hours) at which PD% equals TLC. For analytes that exhibit a mean PD% below the TLC at any time point, the last time point studied was set as SL. The SL in plasma aliquots for stable biochemical analytes in plasma primary tubes was established by verifying that the mean PD% at 96 hours was below the TLC.

## Results

**Figure 1:**
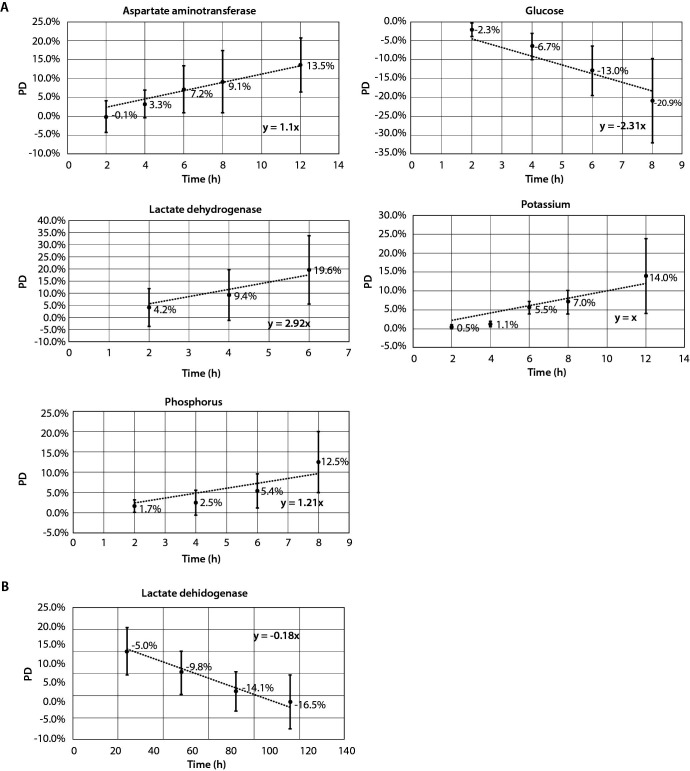
Graphical representation of the mean percentage difference (PD) with respect to storage time for aspartate aminotransferase, glucose, potassium, lactate dehydrogenase and inorganic phosphate in plasma primary samples (A), lactate dehydrogenase in plasma aliquots (B).

Data of the study conducted in plasma primary tubes are shown in Table 1. Among the 29 parameters studied, 24 parameters demonstrated stability in primary plasma tubes within a 48-hour storage period. Hemolysis, icterus and lipemia indices remained consistently stable throughout the study (data not shown). However, the results revealed that five analytes were instable at different time points. Extrapolation of the instability equation at TLC shows a stability limit of 7.9 hours, 2.7 hours, 4.7 hours, 2.9 hours and 6.2 hours for AST, Glc, K, LDH and Phos, respectively. For these analytes, graphical representations of the mean PD% with respect to the storage time are shown in Figure 1A.

**Table 1:** Stability study data in plasma primary tubes

				**Mean PD%**							**SL**
**Parameter**	**CVa (%)**	**CVb (%)**	**TLC (%)**	**2 h**	**4 h**	**6 h**	**8 h**	**12 h**	**24 h**	**48 h**
ADA	8.8	11.7	25.1	- 2.4	2.1	- 1.7	1.6	5.3	2.6	2.3	48 h
Alb	1.6	2.5	4.7	1.6	1.9	2.3	2.8	2.4	1.4	3.5	48 h
ALP	3.5	5.3	10.1	- 2.0	1.9	2.7	1.8	1.0	0.5	- 2.1	48 h
ALT	5.2	10.1	15.3	11.1	10.1	9.5	- 4.6	- 10.7	- 12.7	- 11.1	48 h
AMY	3.1	6.6	9.1	0.1	- 0.8	- 0.1	- 0.1	- 1.8	1.0	1.7	48 h
AST	2.6	9.6	8.7	- 0.1	3.3	7.2	9.1*	13.5*	11.9*	12.7*	7.9 h
Urea	3.9	13.9	12.8	- 0.4	- 1.0	- 1.2	9.6	9.6	-0.2	8.7	48 h
Ca	1.6	1.8	4.6	0.7	0.0	0.9	1.4	1.5	2.4	2.5	48 h
CHOL	1.4	5.3	4.7	1.1	1.5	1.5	2.3	2.5	1.6	3.6	48 h
CK	2.1	15	9.5	0.2	0.8	1.5	2.5	0.6	- 1.1	-1.6	48 h
Cl	1.7	1.1	4.8	0.5	0.0	4.2	4.2	4.6	4.2	3.6	48 h
CREA	3.6	4.5	10.1	3.9	2.4	3.2	0.2	4.6	2.4	0.8	48 h
DBIL	7.1	13.1	20.8	6.0	0.2	- 0.4	- 0.1	2.2	- 4.7	11.1	48 h
GGT	3.1	9.1	9.7	3.2	3.2	7.1	6.2	5.2	5.8	2.7	48 h
Glc	2.1	5	6.3	- 2.3	- 6.7*	- 13.0*	- 20.9*	- 22.1*	- 37.0*	- 66.8*	2.7 h
K	1.5	4.1	4.7	0.5	1.1	5.5*	7.0*	14.0*	43.5*	99.6*	4.7 h
LD	4.0	5.2	11.5	4.2	9.4	19.6*	34.7*	81.2*	38.7*	75.0*	2.9 h
Lip	6.0	9.2	17.2	4.5	5.9	3.9	4.2	NT	5.9	3.7	48 h
Mg	3.9	2.9	11.0	- 1.1	- 1.1	0.3	2.2	2.1	3.7	7.1	48 h
Myoglobin	8.4	17.6	24.9	1.6	- 15.5	- 14.9	- 13.3	- 12.4	NT	- 3.4	48 h
Na	1.1	0.5	3.0	0.4	1.0	1.3	1.8	2.1	1.1	0.0	48 h
Phos	2.3	7.8	7.4	1.7	2.5	5.4	12.5*	16.9*	12.6*	36.1*	6.2 h
fT3	5.2	5	14.6	3.7	5.3	NT	NT	NT	7.7	5.0	48 h
fT4	6.8	4.9	18.9	- 0.3	0.3	NT	NT	NT	3.2	2.0	48 h
TBIL	6.8	20	21.4	- 0.9	0.7	0.2	0.8	1.8	1.0	1.6	48 h
TG	2.0	20	11.4	0.6	0.7	0.7	0.7	0.8	0.1	- 1.2	48 h
TP	1.4	2.6	4.0	0.1	0.9	1.6	2.6	2.1	- 0.9	- 1.9	48 h
TSH	4.5	17.7	15.3	0.5	1.4	NT	NT	NT	- 1.2	1.7	48 h
UA	2.6	8.3	8.3	0.7	0.7	0.6	1.8	1.9	2.0	4.5	48 h
*Mean percentage difference exceeds total limit of change. PD% - percentage difference. CVa - analytical coefficient of variation. CVb - within-subject coefficient of variation. TLC - total limit of change. SL - stability limit. NT - not tested. ADA - adenosine deaminase. Alb - albumin. ALP - alkaline phosphatase. ALT - alanine aminotransferase. AMY - amylase. AST - aspartate aminotransferase. Ca - calcium. CHOL - total cholesterol. CK - creatinine kinase. Cl - chloride. CREA - creatinine. DBIL - direct bilirubin. GGT - gamma-glutamyltransferase. Glc - glucose. K - potassium. LD - lactate dehydrogenase. Lip - lipase. Mg - magnesium. Na - sodium. Phos - inorganic phosphate. fT3 - triiodothyronine-free. fT4 - thyroxine-free. TBIL - total bilirubin. TG - triglycerides. TP - total protein. TSH - thyroid stimulating hormone. UA - uric acid.											

Data of the additional stability study conducted on plasma aliquots are shown in Table 2. Results demonstrated that all parameters were stable for 96 hours, except LD that was stable for 63 hours. Graphical representation of the mean PD% with respect to the storage time for LD is shown in Figure 1B.

**Table 2:** Stability study data in plasma aliquots

		**Mean PD%**				**SL**
**Parameter**	**TLC (%)**	**24 h**	**48 h**	**72 h**	**96 h**
ADA	25.1	NT	NT	NT	6.7	96 h
Alb	4.7	NT	NT	NT	- 1.1	96 h
ALP	10.1	NT	NT	NT	- 4.9	96 h
ALT	15.3	NT	NT	NT	- 11.7	96 h
AMY	9.1	NT	NT	NT	- 0.6	96 h
AST	8.7	1.0	- 1.0	- 2.6	- 2.0	96 h
Urea	12.8	NT	NT	NT	2.1	96 h
Ca	4.6	NT	NT	NT	- 0.5	96 h
CHOL	4.7	NT	NT	NT	0.6	96 h
CK	9.5	NT	NT	NT	- 0.1	96 h
Cl	4.8	NT	NT	NT	0.8	96 h
CREA	10.1	NT	NT	NT	0.4	96 h
DBIL	20.8	NT	NT	NT	- 2.4	96 h
GGT	9.7	NT	NT	NT	2.4	96 h
Glc	6.3	- 1.6	- 1.7	- 1.0	- 0.3	96 h
K	4.7	1.2	- 0.7	- 1.1	- 0.8	96 h
LD	11.5	- 5.0	- 9.8	- 14.1*	- 16.5*	63 h
Lip	17.2	NT	NT	NT	2.7	96 h
Mg	11.0	NT	NT	NT	2.0	96 h
Myoglobin	24.9	NT	NT	NT	2.0	96 h
Na	3.0	NT	NT	NT	-0.5	96 h
Phos	7.4	0.5	0.2	1.5	3.2	96 h
fT3	14.6	NT	NT	NT	-3.6	96 h
fT4	18.9	NT	NT	NT	3.8	96 h
TBIL	21.4	NT	NT	NT	3.6	96 h
TG	11.4	NT	NT	NT	1.2	96 h
TP	4.0	NT	NT	NT	-2.0	96 h
TSH	15.3	NT	NT	NT	1.4	96 h
UA	8.3	NT	NT	NT	1.2	96 h
*Mean percentage difference exceeds total limit of change. PD% - percentage difference. TLC - total limit of change. SL - stability limit. NT - not tested. ADA - adenosine deaminase. Alb - albumin. ALP - alkaline phosphatase. ALT - alanine aminotransferase. AMY - amylase. AST - aspartate aminotransferase. Ca - calcium. CHOL - total cholesterol. CK - creatinine kinase. Cl - chloride. CREA - creatinine. DBIL - direct bilirubin. GGT - gamma-glutamyltransferase. Glc - glucose. K - potassium. LD - lactate dehydrogenase. Lip - lipase. Mg - magnesium. Na - sodium. Phos - inorganic phosphate. fT3 - triiodothyronine-free. fT4 - thyroxine-free. TBIL - total bilirubin. TG - triglycerides. TP - total protein. TSH - thyroid stimulating hormone. UA - uric acid.						

## Discussion

The results of the study revealed different stabilities limits in plasma depending on whether primary or aliquot samples were stored. In primary plasma tubes, five parameters showed stability below 48 hours, indicating that the period for requesting add-on testing in these samples must be carefully controlled to avoid false analytical results. Our findings are aligned with those of prior research indicating that certain analytes are significantly influenced due to prolonged plasma-cell interaction (*5*, *6*). This interaction leads to cellular consumption of the analyte (*i.e.* Glc) or the release of intracellular constituents (*i.e*. AST, LD, Phos and K). The results of the study in plasma aliquots proved to overcome these limitations, even allowing to extend the stability limit of all the analytes studied. Therefore, we implemented a postanalytical aliquoting procedure for all STAT samples. This procedure involves the automatic separation of plasma, being the resultant aliquot automatically stored in the postanalytical refrigerator for four days. This approach allowed us to integrate the storing of STAT samples into the overall laboratory workflow after automation. So that, currently, in our core laboratory we allow add-on orders of 28 analytes in plasma (STAT) samples in the following 96 hours after the first analysis. In case of LD, the laboratory information system enforces an electronic rule to automatically reject add-on testing after 63 hours.

One of the limitations of this study is that it was focused exclusively on one type of specimen and also that stability limits for plasma samples with other anticoagulants or plasma primary tubes with gel separator were not assessed. Additionally, the stability of plasma samples with hemolysis, icterus and lipemia interferences was not evaluated. Another limitation is that it was not possible to simultaneously collect, from the same patient, as many samples as the defined time points for the stability study. Moreover, conducting duplicate measurements for each time point was not feasible due to insufficient plasma volume available to perform all measurements. Nevertheless, in an effort to mitigate the impact of temperature and evaporation on these samples during the analysis, special care was taken to minimize the duration that samples were kept outside the refrigerator.

This study is valuable in defining the maximum allowed period for add-on testing in plasma samples for the majority of biochemical analytes ordered in an emergency context. Data provided could be useful for other clinical laboratories in which plasma samples are stored refrigerated for extended periods, to set stability limits either in primary tubes or aliquots.

## Data Availability

The data generated and analyzed in the presented study are available from the corresponding author on request.
